# Effects of variations in access to care for children with atopic dermatitis

**DOI:** 10.1186/s12895-020-00114-x

**Published:** 2020-12-20

**Authors:** Elaine C. Siegfried, Amy S. Paller, Paola Mina-Osorio, Francis Vekeman, Mandeep Kaur, Usha G. Mallya, Julie Héroux, Raymond Miao, Abhijit Gadkari

**Affiliations:** 1 Department of Pediatrics, Division of Dermatology, Saint Louis University and Cardinal Glennon Children’s Hospital, St. Louis, MO USA; 2 Departments of Dermatology and Pediatrics, Northwestern University Feinberg School of Medicine, Chicago, IL USA; 3 Health Economics and Outcomes Research, Medical Affairs, Regeneron Pharmaceuticals, Inc., 777 Old Saw Mill River Road, Tarrytown, NY 10591 USA; 4 StatLog, Inc., Montreal, Quebec Canada; 5 Sanofi, Cambridge, MA USA; 6 Sanofi, Bridgewater, NJ USA

**Keywords:** Atopic dermatitis, Atopic eczema, Children, Access to care, Medicaid, Private insurance, Emergency department reliance

## Abstract

**Background:**

An estimated 50% of children in the US are Medicaid-insured. Some of these patients have poor health literacy and limited access to medications and specialty care. These factors affect treatment utilization for pediatric patients with atopic dermatitis (AD), the most common inflammatory skin disease in children. This study assesses and compares treatment patterns and healthcare resource utilization (HCRU) between large cohorts of Medicaid and commercially insured children with AD.

**Methods:**

Pediatric patients with AD were identified from 2 large US healthcare claims databases (2011–2016). Included patients had continuous health plan eligibility for ≥6 months before and ≥12 months after the first AD diagnosis (index date). Patients with an autoimmune disease diagnosis within 6 months of the index date were excluded. Treatment patterns and all-cause and AD-related HCRU during the observation period were compared between commercially and Medicaid-insured children.

**Results:**

A minority of children were evaluated by a dermatology or allergy/immunology specialist. Several significant differences were observed between commercially and Medicaid-insured children with AD. Disparities detected for Medicaid-insured children included: comparatively fewer received specialist care, emergency department and urgent care center utilization was higher, a greater proportion had asthma and non-atopic morbidities, high- potency topical corticosteroids and calcineurin inhibitors were less often prescribed, and prescriptions for antihistamines were more than three times higher, despite similar rates of comorbid asthma and allergies among antihistamine users. Treatment patterns also varied substantially across physician specialties.

**Conclusions:**

Results suggest barriers in accessing specialty care for all children with AD and significant differences in management between commercially and Medicaid-insured children. These disparities in treatment and access to specialty care may contribute to poor AD control, especially in Medicaid-insured patients.

**Supplementary Information:**

**Supplementary information** accompanies this paper at 10.1186/s12895-020-00114-x.

## Background

An estimated 50% of children in the US are Medicaid-insured [1, 2]. Access to care for patients enrolled in Medicaid programs is an ongoing concern in the United States (US) [3]. Several studies have shown that patients enrolled in Medicaid are less likely to gain outpatient access to specialty providers [4–7]. A number of factors, including unfavorable fee-for- service reimbursement, longer wait times for payments, and higher clinic non-attendance rates, contribute to the dearth of specialists accepting Medicaid patients [5, 8].

Skin disease is very common in children, prompting up to 30% of all primary care pediatric visits [9]. Atopic dermatitis (AD), a chronic inflammatory skin disease characterized by eczematous lesions and intense pruritus [10–13], is the most common inflammatory skin disease in children, with an estimated prevalence in the United States of about 11–13% among children less than 18 years of age [14, 15]. Up to one-third of these patients are estimated to have moderate-to-severe disease [16], along with a higher risk of atopic and non-atopic morbidities compared with children without AD [13, 17–19]. The burden of AD is substantial, especially in children with moderate-to-severe disease and their caregivers. Chronic sleep disturbance related to persistent pruritus profoundly affects daily functioning, quality of life (QoL), and psychosocial health [20–26]. AD in children is also associated with poorer performance in school, difficulties forming social relationships and participating in sports, and increased rates of anxiety, depression, and even suicidal ideation [20, 22, 24].

While there are currently no treatment guidelines for pediatric AD, the standard of care includes a combination of maintenance skin care and topical medication to prevent and treat flares [27]. Topical medication options include corticosteroids (TCS), calcineurin inhibitors (TCI), and a PDE4 inhibitor. Until the recent approval of dupilumab in adolescents (12–17 years old) with moderate-to-severe AD (March 2019), treatment options among pediatric patients with AD whose disease was not adequately controlled with topical therapies were limited [28]. In fact, for patients with moderate-to-severe AD with uncontrolled symptoms, phototherapy, oral immunosuppressants, and systemic corticosteroids (SCS) are often used off-label, despite potential side effects [29].

Management of AD has been observed to vary greatly across physician specialties [30]. Diagnosis and follow-up care are usually provided by the primary healthcare providers, most often pediatricians and family practitioners [31, 32]. However, primary care training does not include requirements for care of pediatric skin disease, and most trainees have limited exposure to dermatology specialists [9, 33]. Current recommendations for managing pediatric AD suggest dermatology referral for patients with moderate-to-severe and/or refractory, poorly controlled, or generalized AD [34, 35]. Pediatric dermatologists are most experienced in the evaluation and treatment of children with AD, but their limited workforce makes subspecialist access challenging [36].

Restricted access for pediatric Medicaid-insured patients impacts referral to subspecialty care and utilization patterns, particularly for patients needing dermatologic care. A minority of children are successful in obtaining a new patient dermatology appointment [5, 37, 38]. The relapsing nature of AD often prompts patients to seek same-day evaluation at urgent care centers and emergency departments (EDs). These visits often prompt a specialty clinic referral [39], but the outcome of this tactic is unclear.

Although the challenges related to obtaining specialist evaluation and treatment for Medicaid patients are well documented, no study has specifically investigated the global healthcare disparities for children with AD. Understanding and addressing access disparity in pediatric patients with AD is especially critical given the significant healthcare burden for patients, as well as their caregivers. The objective of this study is to assess and compare treatment patterns and healthcare resource utilization (HCRU) of two large cohorts of Medicaid-insured and commercially insured children with AD.

## Methods

This retrospective cohort analysis relied on administrative medical and pharmacy claims from the IBM® MarketScan® Commercial Database and the Multi-State Medicaid Database covering the period from January 1, 2011 to December 31, 2016. Both databases include de-identified patient-level claims and comply with the Health Insurance Portability and Accountability Act (HIPAA) of 1996. Institutional review board approval was not necessary for this study.

Pediatric patients with AD were identified using the following criteria: (1) ≥1 medical claim with a diagnosis of AD (International Classification of disease, ninth revision [ICD-9] code 691.8; ICD-10 codes L20.x), (2) less than 18 years of age on the first observed AD diagnosis (defined as the index date), and (3) continuous health plan eligibility ≥6 months pre-index date (baseline period; up to 6 months for infants <1 year of age) and ≥12 months post-index date. Patients who received a diagnosis of an autoimmune condition (listed in Table 1) during the baseline period or on the index date were excluded. This criterion was applied to help exclude use of treatments of interest for conditions other than AD. The observation period spanned the index date to the end of health plan continuous eligibility or end of data availability, whichever occurred first.

**Table 1:** List of Diagnosis Codes of Autoimmune Conditions Excluded

Code	Type of code	Description
V42.1, V42.0, V42.7, V42.2	ICD-9	Organ transplant (liver, kidney, heart)
Z94.0, Z94.1, Z94.4, Z95.3	ICD-10	Organ transplant (liver, kidney, heart)
710.0	ICD-9	Systematic lupus erythematosus
696.1	ICD-9	Psoriasis
L40.0, L40.1, L40,2, L40.3, L40.4, L40.8	ICD-10	Psoriasis
714.xx	ICD-9	Rheumatoid arthritis
555.9	ICD-9	Crohn’s disease
340.xx	ICD-9	Multiple sclerosis
G35	ICD-10	Multiple sclerosis
704.01	ICD-9	Alopecia areata
L63	ICD-10	Alopecia areata
358.00, 358.01	ICD-9	Myasthenia gravis
G70.00, G70.01	ICD-10	Myasthenia gravis
135	ICD-9	Sarcoidosis
D86.9	ICD-10	Sarcoidosis
582.1	ICD-9	Focal segmental glomerulosclerosis
N03.3	ICD-10	Chronic nephritic syndrome with diffuse mesangial proliferative glomerulonephritis
136.1	ICD-9	Behcet’s disease
M35.2	ICD-10	Behcet’s disease
694.4	ICD-9	Pemphigus
L10.0, L10.1, L10.2, L10.4, L10.9	ICD-10	Pemphigus
556.xx	ICD-9	Ulcerative colitis
696	ICD-9	Psoriatic arthritis
L40.54, L40.59	ICD-10	Psoriatic arthritis
720	ICD-9	Ankylosing spondylitis
364.00, 364.3	ICD-9	Noninfectious uveitis
H20.00, H20.9	ICD-10	Noninfectious uveitis
A67.2	ICD-10	Late lesions of pinta
103.2	ICD-9	Late lesions of pinta
N90.89	ICD-10	Other specified noninflammatory disorders of vulva and perineum
624.8	ICD-9	Other specified noninflammatory disorders of vulva and perineum
L93	ICD-10	Lupus erythematosus
695.4	ICD-9	Lupus erythematosus
D68.62	ICD-10	Lupus anticoagulant syndrome
M32	ICD-10	Systemic lupus erythematosus (SLE)
H01.12	ICD-10	Discoid lupus erythematosus of eyelid
373.34	ICD-9	Discoid lupus erythematosus of eyelid
K50	ICD-10	Crohn’s disease [regional enteritis]
K51	ICD-10	Ulcerative colitis
K52.3	ICD-10	Indeterminate colitis
579	ICD-9	Celiac disease
K90.0	ICD-10	Celiac disease
M45	ICD-10	Ankylosing spondylitis
M08.1	ICD-10	Juvenile ankylosing spondylitis
M05	ICD-10	Rheumatoid arthritis with rheumatoid factor
M06	ICD-10	Other rheumatoid arthritis
M08.0	ICD-10	Unspecified juvenile rheumatoid arthritis
M08.2	ICD-10	Juvenile rheumatoid arthritis with systemic onset
M08.3	ICD-10	Juvenile rheumatoid polyarthritis (seronegative)
M08.4	ICD-10	Pauciarticular juvenile rheumatoid arthritis
M08.9	ICD-10	Juvenile arthritis, unspecified

### Outcomes

Baseline characteristics included age, gender, type of healthcare provider seen on the index date, and AD-related comorbidities evaluated during the 6-month baseline period and on the index date. Treatment patterns considered included the number of prescriptions per year, the proportion of patients with ≥1 combination therapy (overlap ≥3 months between ≥2 distinct AD treatments), and the proportion of patients with ≥1 prescription filled for the selected AD medications among patients with at least one treatment for AD during their observation period (treated patients). Medications assumed to be prescribed to treat AD included: TCS, TCI, antihistamines (topical and oral; sedating and non-sedating), montelukast sodium, SCS, immunosuppressants (azathioprine, cyclosporine A, methotrexate, mycophenolate mofetil, interferon gamma), intravenous immunoglobulin (IVIG), and phototherapy. Although topical and oral antibiotics are often prescribed for infected AD, they are also used for various unrelated, common childhood infections, and so were not included. We were unable to assess the use of crisaborole, approved in December 2016, at the end of our available data. Similarly, dupilumab was not included in the list of selected AD treatments, as it was not yet approved for AD in adolescents over the period covered by the data.

Both all-cause and AD-related HCRU were assessed (including inpatient stays, ED visits, outpatient visits, urgent care center visits, and other resources [primarily patient home, independent laboratory, and other unlisted facilities]). AD-related HCRU was defined as encounters with a diagnosis of AD, an AD-related condition (ICD-9 codes 690.8, 692.9, 705.81; ICD-10 codes L25.9, L30.0, L30.1, L30.3, L30.8, L30.9), a selected skin infection (ICD-9 codes 041.10, 054.0, 686.09, 686.8, 686.9; ICD-10 codes B00.0, B95.8, L08.08, L08.9), or an IVIG or phototherapy procedure. Emergency department reliance (EDR), defined as the ratio of ED and urgent care visits divided by the sum of all ambulatory visits (urgent care + outpatient + ED), was also assessed. The EDR allows distinguishing between patients with frequent ED or urgent care episodes due to an increased need for care, and patients who rely on ED or urgent care center visits to gain access to other outpatient resources. The proportion of patients with high EDR – defined as the percentage of ambulatory visits occurring in the ED setting greater than 33% (i.e., EDR >0.33) – was also evaluated. The cutoff value defining high EDR was determined in accordance with the findings of previous studies [40–42]. Treatment patterns, HCRU, and EDR were evaluated during the entire observation period.

### Statistical analyses

Patient demographics, clinical characteristics, and AD treatment patterns (among treated patients) were summarized and compared between Medicaid-insured patients (Medicaid patients) and commercially insured patients (Commercial patients) using Chi-square and Wilcoxon Mann-Whitney tests for categorical and continuous variables, respectively. An alpha level of 0.05 was used to declare statistical significance. Treatment patterns were stratified by type of healthcare provider seen on the index date (dermatologist, allergist/immunologist [A/I], pediatrician, and “other” provider types [mainly primary care providers (PCPs), nurse practitioners, and acute care providers]) and by age groups (<2 years, 2–5 years, 6–11 years, and 12–17 years). To account for the varying length of observation, HCRU was reported on a per 1000 patient-year basis. Incidence rate ratios (IRR) of HCRU between Medicaid and Commercial patients were estimated using generalized linear models with a log link and negative binomial distribution, adjusting for baseline demographics and clinical characteristics (Table 2). All analyses were performed using SAS version 9.4 (SAS Institute, Cary, NC).

**Table 2:** Demographic and Clinical Characteristics of Patients with AD

	Medicaid *n* = 268,580	Commercial *n* = 338,678	***P***-value
**Demographics**			
Age, mean ± SD | median	5.1 ± 4.9 | 4.0	5.6 ± 5.2 | 4.0	<0.001*
Age category, n (%)			
0–1 yr	89,336 (33.3)	106,255 (31.4)	<0.001*
2–5 yr	69,980 (26.1)	82,290 (24.3)	<0.001*
6–11 yr	73,713 (27.4)	91,400 (27.0)	<0.001*
12–17 yr	35,551 (13.2)	58,733 (17.3)	<0.001*
Male, n (%)	133,153 (49.6)	174,839 (51.6)	<0.001*
**Comorbidities during the 6-month baseline period**^**a**^			
**Patients with ≥1 selected comorbidity, n (%)**	**144,397 (53.8)**	**144,707 (42.7)**	<0.001*
Patients with ≥1 atopic comorbidity, n (%)	89,077 (33.2)	88,135 (26.0)	<0.001*
Allergic conjunctivitis	20,170 (7.5)	22,235 (6.6)	<0.001*
Allergic rhinitis	54,665 (20.4)	50,872 (15.0)	<0.001*
Allergic urticaria	1762 (0.7)	3036 (0.9)	<0.001*
Asthma	40,313 (15.0)	33,038 (9.8)	<0.001*
Chronic rhinosinusitis	4593 (1.7)	5100 (1.5)	<0.001*
Eosinophilic esophagitis	140 (0.1)	331 (0.1)	<0.001*
Food allergy	2928 (1.1)	6644 (2.0)	<0.001*
Nasal polyps	65 (0.0)	94 (0.0)	0.395
**Patients with ≥ 1 other selected comorbidity, n (%)**	**82,491 (30.7)**	**75,381 (22.3)**	<0.001*
Anxiety	2890 (1.1)	3724 (1.1)	0.380
Attention deficit hyperactivity disorder	13,802 (5.1)	6176 (1.8)	<0.001*
Autoimmune disorders	870 (0.3)	1050 (0.3)	0.338
Bacterial infections	8417 (3.1)	11,590 (3.4)	<0.001*
Depression	4474 (1.7)	2330 (0.7)	<0.001*
Fungal infections	46,418 (17.3)	33,992 (10.0)	<0.001*
Obesity	7382 (2.7)	2875 (0.8)	<0.001*
Sleep disorders	1138 (0.4)	444 (0.1)	<0.001*
Viral infections and disorders	13,350 (5.0)	23,506 (6.9)	<0.001*
**Provider type on the index visit, n (%)**			
Dermatology	8545 (3.2)	63,354 (18.7)	<0.001*
Subsequent dermatology or A/I visit after the index visit^b^, n (%)	4523 (52.9)	28,523 (45.0)	<0.001*
Allergy/Immunology	7612 (2.8)	31,626 (9.3)	<0.001*
Subsequent dermatology or A/I visit after the index visit^b^, n (%)	4011 (52.7)	16,050 (50.7)	0.002*
Pediatrics	67,333 (25.1)	166,009 (49.0)	<0.001*
Subsequent dermatology or A/I visit after the index visit^b^, n (%)	6667 (9.9)	24,849 (15.0)	<0.001*
Other	185,090 (68.9)	77,689 (22.9)	<0.001*
Subsequent dermatology or A/I visit after the index visit^b^, n (%)	3752 (2.0)	9392 (12.1)	<0.001*
**Patients who received ≥ 1 AD treatment during the observation period**^**c**^**, n (%)**	240,648 (89.6)	236,836 (69.9)	<0.001*
**Duration of observation period (months), mean ± SD | median**	36.4 ± 16.7 | 34.4	31.3 ± 15.3 | 27.6	<0.001*

Notes

*AD* atopic dermatitis, *SD* standard deviation, *A/I* allergist/immunologist

^a^The baseline period includes the index date. For infants, the baseline period includes up to 6 months of follow-up

^b^Percentages calculated out of the total number of patients with each provider type on the index date

^c^Patients considered in the analysis of treatment patterns

*P-value <0.05. *P*-values were calculated using Chi-square tests for categorical variables, and Wilcoxon Mann-Whitney test for continuous variables

## Results

### Sample

A total of 268,580 and 338,678 patients were identified in the Medicaid and Commercial cohorts, respectively. These relative numbers reflect the sizes of the Commercial and Medicaid samples in the IBM MarketScan databases over the 2011–2016 period, rather than the absolute proportion of children insured by each category.

### Patient characteristics

Medicaid patients were younger than Commercial patients (mean age: 5.1 vs. 5.6; *p* < 0.001; Table 2) and had a longer observation period (median 34.4 months vs. 27.6; *p* < 0.001). These patients were also more likely during the baseline period to have ≥1 atopic comorbidity (33.2% vs. 26.0%; *p* < 0.001), with the most important differences observed for allergic rhinitis (20.4% vs. 15.0%; *p* < 0.001) and asthma (15.0% vs. 9.8%; *p* < 0.001). The proportion of patients with ≥1 other non-atopic comorbidity documented was higher in Medicaid-insured children (30.7% vs. 22.3%; *p* < 0.001), including fungal infection (17.3% vs. 10.0%; *p* < 0.001).

On the index date, the largest subset of patients (43%) were seen by non-specialist providers (other provider types), followed by pediatricians (38%), dermatologists (12%), and A/I (6%) (Table 2). On the index date, Medicaid patients were less likely to be seen by a pediatrician (25.1% Medicaid vs. 49.0% Commercial), a dermatologist (3.2% vs. 18.7%), or an A/I (2.8% vs. 9.3%; *p* < 0.001 for all). However, a greater proportion of Medicaid patients saw a non-specialist provider (other provider types) on the index date (68.9% compared to 22.9% Medicaid). Medicaid patients were also less likely with subsequently visit a dermatologist or an A/I during their observation period, except for those who were seen by a dermatologist or an A/I on their index visit.

### Treatment patterns

During the observation period, Medicaid patients were more likely to be prescribed ≥1 treatment for AD compared with commercially insured patients (89.6% vs. 69.9%; *p* < 0.001). All treatment patterns were assessed and compared in this sample of treated patients with Medicaid versus Commercial coverage.

Compared with commercially insured patients, those covered by Medicaid had more prescriptions filled per year (mean: 3.3 vs. 2.0; patients with >3 prescriptions per year: 32.1% vs. 18.1%; *p* < 0.001; Table 3) and were more likely to receive combination therapy (40.5% vs. 19.5%; *p* < 0.001). Over the study period, 87.6% and 86.1% of Medicaid and commercially insured patients received ≥1 topical treatment, respectively. Medicaid patients were more likely to receive low- potency TCS (41.7% vs. 31.2%), and less likely to receive high- potency TCS (12.9% vs. 16.5%) or TCI (3.3% vs. 7.4%; *p* < 0.001 for all). These treatment differences were generally the most pronounced in patients 6–11 years if age and 12–17 years of age (see Supplementary Table 1, Additional file 1).

**Table 3:** Treatment Patterns of Patients with AD (Entire Observation Period)

	Medicaid *n* = 240,648	Commercial *n* = 236,836	***P***-value
**Filled prescriptions per year, mean ± SD | median**	**3.3 ± 4.3 | 1.8**	**2.0 ± 2.8 | 1.0**	<0.001*
≤1 prescription, n (%)	75,572 (31.4)	120,996 (51.1)	<0.001*
2–3 prescriptions, n (%)	87,804 (36.5)	73,057 (30.8)	<0.001*
>3 prescriptions, n (%)	77,272 (32.1)	42,783 (18.1)	<0.001*
**Combination therapy with ≥2 distinct AD treatments, n (%)**	**97,366 (40.5)**	**46,171 (19.5)**	<0.001*
**Topical treatments, n (%)**	**210,857 (87.6)**	**203,975 (86.1)**	<0.001*
Topical antihistamines	24 (0.0)	55 (0.0)	<0.001*
Any topical corticosteroids (TCS)	210,094 (87.3)	200,975 (84.9)	<0.001*
TCS low potency	100,439 (41.7)	74,010 (31.2)	<0.001*
TCS medium potency	158,484 (65.9)	147,297 (62.2)	<0.001*
TCS high potency	31,084 (12.9)	38,999 (16.5)	<0.001*
Topical calcineurin inhibitors (TCI)	7998 (3.3)	17,579 (7.4)	<0.001*
**Systemic antihistamines**^**a**^	**170,511 (70.9)**	**51,817 (21.9)**	<0.001*
≥1 sedating antihistamine	78,319 (32.5)	37,094 (15.7)	<0.001*
**Systemic corticosteroids (SCS)**^**b**^	**54,878 (22.8)**	**59,626 (25.2)**	<0.001*
**Any systemic immunosuppressants (IMM)**	**476 (0.2)**	**630 (0.3)**	<0.001*
Azathioprine	54 (0.0)	93 (0.0)	<0.001*
Cyclosporine A	85 (0.0)	167 (0.1)	<0.001*
Interferon gamma	0 (0.0)	1 (0.0)	–
Methotrexate	261 (0.1)	339 (0.1)	<0.001*
Mycophenolate mofetil	140 (0.1)	136 (0.1)	0.914
**Intravenous immunoglobulin (IVIG)**	**2339 (1.0)**	**2784 (1.2)**	<0.001*
**Montelukast sodium**^**c**^	**37,719 (15.7)**	**39,810 (16.8)**	<0.001*
**Phototherapy, n (%)**	**416 (0.2)**	**522 (0.2)**	<0.001*

Notes

*AD* atopic dermatitis, *SD* standard deviation

^a^The proportion of patients without comorbid asthma or allergies was 60.3% and 67.5% among Medicaid and Commercial patients prescribed systemic antihistamines, respectively

^b^The proportion of patients without comorbid asthma or allergies was 45.8% and 56.4% among Medicaid and Commercial patients prescribed SCS, respectively

^c^The proportion of patients without comorbid asthma or allergies was 35.3% and 46.9% among Medicaid and Commercial patients prescribed montelukast sodium, respectively

*P-value< 0.05. *P*-values were calculated using Chi-square tests for categorical variables, and Wilcoxon Mann-Whitney test for continuous variables

Compared with commercially insured patients, the proportion of Medicaid-insured patients who received at least 1 prescription for a systemic antihistamine was more than three times higher (70.9% vs. 21.9%; *p* < 0.001). Among those who were prescribed antihistamines, almost half of Medicaid patients and almost two-thirds of commercially insured patients were prescribed a sedating antihistamine (Table 3). Among patients prescribed oral antihistamines in both cohorts, most did not have comorbid asthma or allergies during the baseline period (Medicaid: 60.3%; Commercial: 67.5%; *p* < 0.001). A slightly smaller proportion of Medicaid patients received SCS (22.8% vs. 25.2%; *p* < 0.001).

Among patients with a dermatologist visit on the index date, Medicaid patients were more likely to be prescribed high- potency TCS (34.5% vs. 27.1%; *p* < 0.001) and SCS (27.0% vs. 25.5%; *p* = 0.006) (see Supplementary Table 2, Additional file 1), but less likely to be prescribed TCI (11.8% vs. 13.6%; *p* < 0.001) than Commercial patients. Among patients who saw an A/I or a pediatrician on the index date, Medicaid patients were more likely to be prescribed a SCS (A/I: 47.9% vs. 42.5%; *p* < 0.001, pediatrician: 21.0% vs. 20.2%; *p* < 0.001) (see Supplementary Table 2, Additional file 1). However, among patients who saw a provider other than a dermatologist, A/I, or pediatrician on the index date (68.6% vs. 22.9% of Medicaid and Commercial patients, respectively), Medicaid patients were less likely to be prescribed high- potency TCS (12.6% vs. 16.5%), TCI (2.9% vs. 6.9%), or SCS (22.2% vs. 27.5%; *p* < 0.001 for all). Regardless of the payor type, high- potency TCS and TCI were most often prescribed by dermatologists and montelukast sodium by A/I.

Significant differences in antihistamine prescribing patterns were also observed across provider types. Systemic antihistamines were most often prescribed to patients who saw a non-specialist provider (other providers) on the index date – prescribed in more than half of these patients. Differences were also observed with regard to the proportion of sedating antihistamines prescribed (Table 4). Among patients who were prescribed a systemic antihistamine, sedating antihistamines were prescribed in 72.9% of patients who saw a dermatologist on the index date, compared with around 50% for those who saw the other types of provider. Among commercially insured patients who were prescribed systemic antihistamines, the majority received sedating antihistamines, reaching up to 80% of patients among those who saw a dermatologist on the index date.

**Table 4:** Antihistamine Utilization (Entire Observation Period) - Stratified by Provider Type

	Dermatologist	A/I	Pediatrics	Other
**Number of patients, n (%)**	**58,758 (12.3)**	**29,994 (6.3)**	**169,455 (35.5)**	**219,277 (45.8)**
**Systemic antihistamine use, n (%)**	**17,195 (29.3)**	**13,146 (43.8)**	**63,904 (37.7)**	**128,083 (58.4)**
Non-sedating antihistamine only^a^, n (%)	4651 (27.0)	6556 (49.9)	32,424 (50.7)	63,284 (49.4)
≥1 sedating antihistamine, n (%)	12,544 (72.9)	6590 (50.1)	31,480 (49.3)	64,799 (50.6)
**Medicaid-insured patients, n (%)**	**8160**	**7144**	**60,268**	**165,076**
**Systemic antihistamine use, n (%)**	**5933 (72.7)**	**6123 (85.7)**	**42,416 (70.4)**	**116,039 (70.3)**
Non-sedating antihistamine only^a^, n (%)	2467 (41.6)	3897 (63.6)	25,852 (60.9)	59,976 (51.7)
≥1 sedating antihistamine, n (%)	3466 (58.4)	2226 (36.4)	16,564 (39,1)	56,063 (48.3)
**Commercially insured patients, n (%)**	**50,598**	**22,850**	**109,187**	**54,201**
**Systemic antihistamine use, n (%)**	**11,262 (22.3)**	**7023 (30.7)**	**21,488 (19.7)**	**12,044 (22.2)**
Non-sedating antihistamine only^a^, n (%)	2184 (19.4)	2659 (37.9)	6572 (30.6)	3308 (27.5)
≥1 sedating antihistamine, n (%)	9078 (80.6)	4364 (62.1)	14,916 (69.4)	8736 (72.5)

A/I Allergist/Immunologist, Rx Prescription

^a^Non-sedating antihistamines do not require Rx, but some Medicaid plans will cover the cost with Rx

### Healthcare resource utilization

**Fig. 1:**
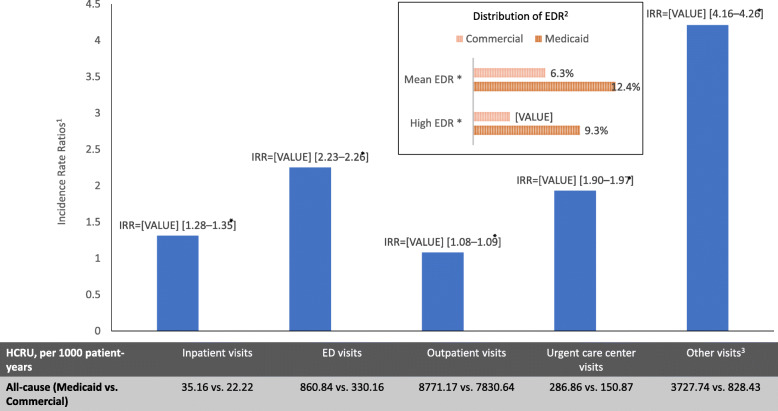
Incidence Rate Ratios of All-Cause HCRU of Patients with AD – Medicaid vs. Commercial (Reference Group). Notes: AD: atopic dermatitis; ED: emergency department; EDR: emergency department reliance; HCRU: healthcare resource utilization. 1. Estimated using generalized linear models with a log link and negative binomial distribution, adjusting for baseline demographics and clinical characteristics. 2. High EDR is defined with a percentage of ambulatory visits occurring in the ED of at least 33% (i.e., EDR >0.33). 3. Includes primarily patient home, independent laboratory, and other unlisted facilities. *P-value <0.05

Although all-cause HCRU was higher among Medicaid patients than among Commercial patients for all types of encounter (adjusted IRR; inpatient: 1.31; ED: 2.25; outpatient: 1.08; urgent care centers: 1.93; other: 4.21; all *p* < 0.001; Fig. 1), the rate of AD-related outpatient visits was lower (visits per 1000 patient years: 849.72 Medicaid vs. 899.22 Commercial; adjusted IRR: 0.93; *p* < 0.001, results not shown). A larger minority of Medicaid patients had AD-related ED visits (10.8% Medicaid, 3.2% Commercial), with an adjusted rate more than twice as high (visits per 1000 patient years: 49.43 vs. 14.81; adjusted IRR: 2.37; *p* < 0.001, results not shown). Mean EDR was 12.4% for Medicaid patients versus 6.3% for Commercial patients (*p* < 0.001; Fig. 1), reflecting a greater proportion of high EDR (i.e., EDR >0.33) among Medicaid patients (9.3% vs. 3.2%; *p* < 0.001).

## Discussion

Using administrative healthcare claims data, this study aimed to compare real-world patterns of care, medications prescribed, and HCRU between two large cohorts of children with AD covered by Medicaid and by Commercial insurance plans. Access to medical care, and especially subspecialty care, for pediatric patients is an issue that is well recognized, but not well studied. Most publications have used surveys of either providers or caregivers for broad therapeutic areas [3, 17, 19, 24]. Few reports have focused on variations in treatment patterns of pediatric patients with AD observed across different providers [5–7, 37, 39].

This study provides a unique portrait of patterns of care for AD derived from databases including large samples of Medicaid- and commercially insured children. Moreover, analyses of treatments and HCRU stratified by provider type provide additional insights about the nature of potential healthcare disparities.

The type of clinician seen by patients for their initial AD-related visit varied greatly between Medicaid and Commercial patients. Although a small proportion of all pediatric patients, regardless of insurance type, were seen by a dermatologist or A/I on their index visit, the proportion of Medicaid patients who did was significantly lower than for commercially insured patients (dermatologist: 3.2% vs. 18.7%; A/I: 2.8% vs. 9.3%). Furthermore, Medicaid patients were three times as likely to be seen by a provider type other than a dermatologist, allergist, or pediatrician, compared with Commercial patients (68.9% vs. 22.9%). These results support, but exceed, those of a retrospective study using data from the 2008 to 2012 Medical Expenditure Panel Survey, documenting a <50% likelihood of receiving a diagnosis for a skin-related disease from a dermatologist, for Medicaid-insured compared with privately insured individuals [43]. The large proportion of Medicaid patients seen by other provider types may reflect evaluation/management of Medicaid patients by a non-specialist provider in an ED or urgent care setting.

Other studies have also documented low acceptance rate of new Medicaid patients by specialists. One 2004 survey of 612 dermatologists in the US found acceptance rates to be much lower for Medicaid patients than for privately insured patients (32% vs. 87%) [7]. In a 2011 US study focused on access to ambulatory dermatology care for new pediatric patients with AD, the average Medicaid acceptance rate was 19% among 471 dermatologists surveyed [37]. Our larger databases included even fewer referrals to specialists following the initial AD-related visit. Among patients initially seen by a provider type classified as “other”, only 2% of Medicaid patients versus 12% of Commercial patients had a subsequent visit with a dermatologist or an A/I. This is particularly surprising given the significantly greater proportion of Medicaid versus Commercial patients with extracutaneous morbidities, suggesting a greater need for subspecialty care in this cohort.

The majority of Medicaid patients were seen by other types of providers (68.9% vs. 22.9% Commercial), mainly PCPs, nurse practitioners, and acute care providers, all of whom have been found to approach treatment of AD with different prescribing patterns [30]. High- potency TCS and TCI were most often prescribed for patients who saw a dermatologist on their index visit. This finding supports greater comfort using higher potency agents among dermatologists, who may be more familiar with the principles of topical treatment and the low risk of side effects when these medications are used as recommended [30, 44]. Overall, Medicaid-insured children were less likely to be prescribed high- potency TCS, SC, and TCI. Lower TCI utilization among Medicaid patients may be related to formulary restrictions and more limited access to dermatologists, the provider type most often prescribing TCI.

Stratification analyses by provider type showed that, for the small proportion of Medicaid-insured patients who saw a dermatologist or A/I on the index date, a similar or even higher proportion of patients received high- potency TCS and SCS compared with commercially insured patients seen by the same type of specialists. This suggests that fewer disparities between Medicaid and commercially insured patients remain when patients are managed by dermatologists or A/I. These results also support the fact that the limited access to dermatologists for Medicaid patients could, in part, explain their overall lower utilization of higher potency treatments. The higher proportion of patients who were prescribed high- potency TSC and SCS among Medicaid patients seen by a dermatologist may potentially be explained by their heavier comorbid profile and, given their more limited access to specialists, the fact that only the most severe AD cases are referred to/managed by dermatologists [5, 7, 37, 38].

Oral antihistamines are regularly used in AD, despite their reported lack of efficacy beyond promoting sleep and their potential adverse effects [45, 46]. Our results reflected this trend. Although antihistamines are sometimes prescribed to treat atopic comorbidities (e.g., urticaria and allergic rhinitis) and sedating antihistamines to treat AD for their soporific effect [47–49], the difference in the utilization of all antihistamines by Medicaid- and commercially insured patients was striking (70.9% vs. 21.9%) – despite similar rates of comorbid asthma or allergies (60.3% and 67.5%). Moreover, the proportion of Medicaid patients receiving sedating antihistamines was more than twice that of Commercial patients (32.5% vs. 15.7%; of whom 64.9% and 71.5%, respectively, did not have comorbid asthma/allergies). Two common over-the-counter (OTC) antihistamines, loratadine and diphenhydramine, were prescribed 61 and 75 times more often to Medicaid- than commercially insured children (28.2% vs. 0.5% and 10.6% vs. 0.1%, respectively). Major factors that could affect antihistamine prescribing include need for a prescription as well as insurance coverage (regardless of prescription-restricted status). Several sedating antihistamines are available by prescription only; however, some are not, such as diphenhydramine, which is available without a prescription. All non-sedating antihistamines are available without a prescription, but many Medicaid plans and some Commercial plans will cover the cost of OTC antihistamines with a prescription, including loratadine. Accordingly, OTC antihistamines may be commonly used by commercially insured patients, but not detectable in the database because the costs are not covered by the Commercial health plans but are covered by Medicaid programs. Widespread use of antihistamines by these patients, especially those who are Medicaid-insured, also highlights possible over use. More limited Medicaid formularies that restrict access to many topical corticosteroids and TCIs may contribute to this health disparity.

We also detected a difference in antihistamine prescribing patterns among specialties. The majority of antihistamine prescriptions (72.9%) among patients who saw a dermatologist on the index date were for a sedating product, compared with 50.1% for those who saw an A/I, and 49.3% and 50.6% for those who saw a pediatrician and other provider types. This may be related to better understanding about histamine’s lack of contribution to dermatitis itch and use of sedating antihistamines for their soporific effects. Among the few patients who were treated with immunosuppressants (Medicaid: 0.2%; Commercial: 0.3%), methotrexate was the preferred choice, regardless of the payor type. This is consistent with the results of a survey of pediatric dermatologists in the US and Canada that identified methotrexate as one of the top first- and second-line choices of immunosuppressants [50].

A potential explanation for the overall higher HCRU of Medicaid versus Commercial patients may be their heavier comorbid profile (atopic comorbidities: 33.2% vs. 26.0%; other comorbidities: 30.7% vs. 22.3%). It may also be the result of disparities in access to care, and more specifically to specialty care. Medicaid patients were found to be more than twice as likely to have an AD-related visit to the ED compared with Commercial patients, a finding corroborated by an analysis of the 2006–2012 Nationwide Emergency Department Sample [51].

The EDR provided further understanding into the differences in ED and urgent care center use between the two populations. Medicaid patients were close to three times as likely to have a high EDR (EDR >0.33) compared with Commercial patients, suggesting that Medicaid patients rely more heavily on ED and urgent care center visits for ongoing care.

Qualitative interviews of 26 specialists and 14 PCPs indicated that pediatric patient referral through the ED was common practice for PCPs in order to obtain access to specialists for patients under public insurance coverage, regardless of the condition [39]. Another study looking at the use of ED among children with AD found that most ED visits were by publicly insured patients [52]. These findings corroborate those of numerous other studies, suggesting a need for improved utilization and convenient access to primary care and specialty outpatient care for Medicaid recipients [53, 54].

A few limitations affected this large administrative healthcare claims analysis. One challenge is related to nonspecific use of the term “eczema”, a larger group of dermatoses that includes AD as well as a wide range of other ICD diagnostic codes [55]. In order to limit our cohort to patients with AD and exclude those with other forms of eczema, only AD-specific ICD codes were used to identify relevant patients. We also conducted sensitivity analyses for a larger cohort that included a broader range of eczema-related diagnostic codes, with similar results. An additional limitation is failure to capture patients with AD who did not seek care for their symptoms, potentially skewing the study sample towards patients with more severe disease. Finally, race information was unavailable for Commercial patients (available only for Medicaid patients) and is thus not included. A growing body of data indicates differences among various racial AD patient groups, including greater severity among Black and Hispanic patients with AD [15]. Thus, some of the differences observed between Medicaid and Commercial patients may be due to disparities in the race distribution among patients in the two samples.

## Conclusion

Results from this claims data analysis comparing two large pediatric AD cohorts, Medicaid- and commercially insured children, indicate that an overall minority of patients were seen by a specialist. Non-specialist providers saw a considerably greater proportion of Medicaid patients, compared with specialists, with dermatologists seeing the very smallest proportion. Accordingly, it was not surprising that Medicaid patients had a higher reliance on ED and urgent care centers, especially for AD-related care, with a rate of ED visits more than twice as high for Medicaid compared with Commercial patients, highlighting the importance of barriers to accessing outpatient and specialist care. Finally, antihistamines were prescribed more than three times more often to Medicaid patients. There is currently no well-established standard-of-care or pediatric-specific guideline accepted by clinicians for AD, and treatment approaches vary greatly across physician specialties. These variations are amplified by disparities in access to specialty care, exacerbating the unmet treatment needs for children with AD. A more consistent and concerted approach is needed to treat this chronic condition. Long-term disease control has the potential to alleviate the direct burden of AD and impede the risk of developing atopic and non-atopic comorbidities and, in turn, may help reduce the utilization of healthcare resources in this patient population.

## Supplementary information

Additional file 1. This file contains two supplementary tables. **Supplementary Table 1.** Reports the treatment patterns of patients with AD stratified by age group. **Supplementary Table 2.** Reports the treatment patterns of patients with AD stratified by provider type. These tables were not included in the main text because they are larger than A4.

## Data Availability

The data that support the findings of this study are available from Truven Health Analytics, but restrictions apply to the availability of these data, which were used under a license agreement for the current study and, accordingly, are not publicly available. Access to the IBM® MarketScan® Commercial Database and the Multi-State Medicaid Database can be requested by contacting Truven Health Analytics.

## Abbreviations

A/I: Allergist/immunologist
AD: Atopic dermatitis
ED: Emergency department
EDR: Emergency department reliance
HCRU: Healthcare resource utilization
IRR: Incidence rate ratio
IVIG: Intravenous immunoglobulin
OTC: Over-the-counter
PCP: Primary care provider
QoL: Quality of life
SCs: Systemic corticosteroids
TCI: Topical calcineurin inhibitors
TCS: Topical corticosteroids
US: United States
