# Supplementary material for: Effects of variations in access to care for children with atopic dermatitis
Source: BMC Dermatol. 2020 Dec 20;20:24. doi: 10.1186/s12895-020-00114-x (PMC7749983; doi:10.1186/s12895-020-00114-x)
Supplement: Supplementary file 1 — This file contains two supplementary tables. Supplementary Table 1. Reports the treatment patterns of patients with AD stratified by age group. Supplementary Table 2. Reports the treatment patterns of patients with AD stratified by provider type. These tables were not included in the main text because they are larger than A4. [file 12895_2020_114_MOESM1_ESM.docx]

**Supplementary Table 1. Treatment Patterns of Patients with AD (Entire Observation Period) - Stratified by Age Group**

|  | **0-1 yr** | | | **2-5 yr** | | | **6-11 yr** | | | **12-17 yr** | | |
| --- | --- | --- | --- | --- | --- | --- | --- | --- | --- | --- | --- | --- |
|  | **Medicaid** | **Commercial** | **P-value** | **Medicaid** | **Commercial** | **P-value** | **Medicaid** | **Commercial** | **P-value** | **Medicaid** | **Commercial** | **P-value** |
|  | n = 77,758 | n = 67,925 |  | n = 63,308 | n = 56,762 |  | n = 67,086 | n = 66,386 |  | n = 32,496 | n = 45,763 |  |
| **Filled prescriptions per year, mean ± SD \| median** | **2.9 ± 3.6 \| 1.7** | **1.9 ± 2.5 \| 1.0** | **<.001*** | **3.3 ± 4.4 \| 1.7** | **2.1 ± 3.0 \| 0.9** | **<.001*** | **3.6 ± 4.9 \| 1.8** | **2.1 ± 3.0 \| 1.0** | **<.001*** | **3.5 ± 4.6 \| 1.8** | **2.0 ± 2.7 \| 1.0** | **<.001*** |
| ≤ 1 prescription, n (%) | 24,506 (31.5%) | 34,860 (51.3%) | <.001* | 20,435 (32.3%) | 29,785 (52.5%) | <.001* | 20,815 (31.0%) | 33,802 (50.9%) | <.001* | 9,816 (30.2%) | 22,549 (49.3%) | <.001* |
| 2-3 prescriptions, n (%) | 29,834 (38.4%) | 21,378 (31.5%) | <.001* | 22,907 (36.2%) | 16,409 (28.9%) | <.001* | 23,267 (34.7%) | 19,892 (30.0%) | <.001* | 11,796 (36.3%) | 15,378 (33.6%) | <.001* |
| > 3 prescriptions, n (%) | 23,418 (30.1%) | 11,687 (17.2%) | <.001* | 19,966 (31.5%) | 10,568 (18.6%) | <.001* | 23,004 (34.3%) | 12,692 (19.1%) | <.001* | 10,884 (33.5%) | 7,836 (17.1%) | <.001* |
| **Combination therapy with ≥2 distinct AD treatments, n (%)** | **24,690 (31.8%)** | **10,883 (16.0%)** | **<.001*** | **27,323 (43.2%)** | **10,705 (18.9%)** | **<.001*** | **31,156 (46.4%)** | **13,921 (21.0%)** | **<.001*** | **14,197 (43.7%)** | **10,662 (23.3%)** | **<.001*** |
| **Topical treatments, n (%)** | **69,377 (89.2%)** | **60,777 (89.5%)** | **0.116** | **55,644 (87.9%)** | **48,406 (85.3%)** | **<.001*** | **57,946 (86.4%)** | **55,628 (83.8%)** | **<.001*** | **27,890 (85.8%)** | **39,164 (85.6%)** | **0.333** |
| Topical antihistamines | 8 (0.0%) | 9 (0.0%) | 0.602 | 5 (0.0%) | 15 (0.0%) | 0.013* | 7 (0.0%) | 15 (0.0%) | 0.084 | 4 (0.0%) | 16 (0.0%) | 0.051 |
| Any topical corticosteroids (TCS) | 69,301 (89.1%) | 60,458 (89.0%) | 0.475 | 55,455 (87.6%) | 47,738 (84.1%) | <.001* | 57,628 (85.9%) | 54,468 (82.0%) | <.001* | 27,710 (85.3%) | 38,311 (83.7%) | <.001* |
| TCS low potency | 42,798 (55.0%) | 31,327 (46.1%) | <.001* | 25,894 (40.9%) | 17,926 (31.6%) | <.001* | 22,920 (34.2%) | 15,919 (24.0%) | <.001* | 8,827 (27.2%) | 8,838 (19.3%) | <.001* |
| TCS medium potency | 48,811 (62.8%) | 42,027 (61.9%) | <.001* | 42,713 (67.5%) | 35,152 (61.9%) | <.001* | 45,028 (67.1%) | 41,468 (62.5%) | <.001* | 21,932 (67.5%) | 28,650 (62.6%) | <.001* |
| TCS high potency | 7,828 (10.1%) | 6,943 (10.2%) | 0.330 | 7,835 (12.4%) | 7,217 (12.7%) | 0.077 | 9,487 (14.1%) | 11,762 (17.7%) | <.001* | 5,934 (18.3%) | 13,077 (28.6%) | <.001* |
| Topical calcineurin inhibitors (TCI) | 1,719 (2.2%) | 3,438 (5.1%) | <.001* | 2,233 (3.5%) | 3,938 (6.9%) | <.001* | 2,659 (4.0%) | 5,558 (8.4%) | <.001* | 1,387 (4.3%) | 4,645 (10.2%) | <.001* |
| **Systemic antihistamines^1^** | **53,591 (68.9%)** | **16,068 (23.7%)** | **<.001*** | **47,158 (74.5%)** | **13,272 (23.4%)** | **<.001*** | **48,228 (71.9%)** | **12,998 (19.6%)** | **<.001*** | **21,534 (66.3%)** | **9,479 (20.7%)** | **<.001*** |
| ≥ 1 sedating antihistamine | 26,507 (34.1%) | 11,026 (16.2%) | <.001* | 21,857 (34.5%) | 9,087 (16.0%) | <.001* | 19,901 (29.7%) | 9,237 (13.9%) | <.001* | 10,054 (30.9%) | 7,744 (16.9%) | <.001* |
| **Systemic corticosteroids (SC)^2^** | **8,451 (10.9%)** | **8,705 (12.8%)** | **<.001*** | **12,185 (19.2%)** | **11,529 (20.3%)** | **<.001*** | **21,135 (31.5%)** | **20,973 (31.6%)** | **0.729** | **13,107 (40.3%)** | **18,419 (40.2%)** | **0.810** |
| **Any systemic immunosuppressants (IMM)** | **73 (0.1%)** | **51 (0.1%)** | **0.220** | **113 (0.2%)** | **105 (0.2%)** | **0.792** | **169 (0.3%)** | **230 (0.3%)** | **0.002*** | **121 (0.4%)** | **244 (0.5%)** | **0.001*** |
| Azathioprine | 4 (0.0%) | 5 (0.0%) | 0.591 | 8 (0.0%) | 2 (0.0%) | 0.084 | 31 (0.0%) | 33 (0.0%) | 0.770 | 11 (0.0%) | 53 (0.1%) | <.001* |
| Cyclosporine A | 20 (0.0%) | 14 (0.0%) | 0.524 | 22 (0.0%) | 27 (0.0%) | 0.272 | 25 (0.0%) | 62 (0.1%) | <.001* | 18 (0.1%) | 64 (0.1%) | <.001* |
| Interferon gamma | 0 (0.0%) | 0 (0.0%) | 1.000 | 0 (0.0%) | 0 (0.0%) | 1.000 | 0 (0.0%) | 1 (0.0%) | - | 0 (0.0%) | 0 (0.0%) | 1.000 |
| Methotrexate | 37 (0.0%) | 23 (0.0%) | 0.198 | 63 (0.1%) | 67 (0.1%) | 0.330 | 92 (0.1%) | 128 (0.2%) | 0.012* | 69 (0.2%) | 121 (0.3%) | 0.145 |
| Mycophenolate mofetil | 19 (0.0%) | 14 (0.0%) | 0.629 | 34 (0.1%) | 23 (0.0%) | 0.295 | 48 (0.1%) | 45 (0.1%) | 0.794 | 39 (0.1%) | 54 (0.1%) | 0.936 |
| **Intravenous immunoglobulin (IVIG)** | **921 (1.2%)** | **930 (1.4%)** | **0.002*** | **651 (1.0%)** | **728 (1.3%)** | **<.001*** | **496 (0.7%)** | **600 (0.9%)** | **<.001*** | **271 (0.8%)** | **526 (1.1%)** | **<.001*** |
| **Montelukast sodium^3^** | **8,059 (10.4%)** | **8,836 (13.0%)** | **<.001*** | **11,695 (18.5%)** | **11,430 (20.1%)** | **<.001*** | **13,169 (19.6%)** | **13,439 (20.2%)** | **0.005*** | **4,796 (14.8%)** | **6,105 (13.3%)** | **<.001*** |
| **Phototherapy, n (%)** | **34 (0.0%)** | **28 (0.0%)** | **0.817** | **109 (0.2%)** | **75 (0.1%)** | **0.077** | **153 (0.2%)** | **193 (0.3%)** | **0.024*** | **120 (0.4%)** | **226 (0.5%)** | **0.010*** |

**Notes:**

AD: Atopic dermatitis; SD: Standard deviation

1. The proportion of Medicaid and Commercial patients without comorbid asthma or allergies was 80.3% and 83.1% for 0-1 yr, 53.8% and 60.1% for 2-5 yr, 47.6% and 55.4% for 6-11 yr, and 53.3% and 67.7% for 12-17 yr.

2. The proportion of Medicaid and Commercial patients without comorbid asthma or allergies was 74.8% and 78.4% for 0-1 yr, 35.4% and 46.1% for 2-5 yr, 37.1% and 46.1% for 6-11 yr, and 50.8% and 64.1% for 12-17 yr.

3. The proportion of Medicaid and Commercial patients without comorbid asthma or allergies was 70.1% and 74.0% for 0-1 yr, 30.2% and 41.8% for 2-5 yr, 22.2% and 35.7% for 6-11 yr, and 25.7% and 41.9% for 12-17 yr.

*P-value<0.05. P-values were calculated using Chi-square tests for categorical variables, and Wilcoxon Mann-Whitney test for continuous variables.

**Supplementary Table 2. Treatment Patterns of Patients with AD (Entire Observation Period) - Stratified by Provider Type**

|  | **Dermatology** | | | **Allergy/Immunology** | | | **Pediatrics** | | | **Other Provider** | | |
| --- | --- | --- | --- | --- | --- | --- | --- | --- | --- | --- | --- | --- |
|  | **Medicaid** | **Commercial** | **P-value** | **Medicaid** | **Commercial** | **P-value** | **Medicaid** | **Commercial** | **P-value** | **Medicaid** | **Commercial** | **P-value** |
|  | n = 8,160 | n = 50,598 |  | n = 7,144 | n = 22,850 |  | n = 60,268 | n = 109,187 |  | n = 165,076 | n = 54,201 |  |
| **Filled prescriptions per year, mean ± SD \| median** | **4.5 ± 5.4 \| 2.6** | **2.1 ± 2.8 \| 1.1** | **<.001*** | **6.8 ± 7.1 \| 4.4** | **3.4 ± 4.1 \| 1.8** | **<.001*** | **3.0 ± 3.9 \| 1.7** | **1.7 ± 2.3 \| 0.9** | **<.001*** | **3.2 ± 4.2 \| 1.7** | **2.0 ± 2.8 \| 1.0** | **<.001*** |
| ≤ 1 prescription, n (%) | 1,867 (22.9%) | 24,276 (48.0%) | <.001* | 980 (13.7%) | 7,738 (33.9%) | <.001* | 19,857 (32.9%) | 61,303 (56.1%) | <.001* | 52,868 (32.0%) | 27,679 (51.1%) | <.001* |
| 2-3 prescriptions, n (%) | 2,649 (32.5%) | 16,849 (33.3%) | 0.136 | 1,754 (24.6%) | 7,129 (31.2%) | <.001* | 22,166 (36.8%) | 32,413 (29.7%) | <.001* | 61,235 (37.1%) | 16,666 (30.7%) | <.001* |
| > 3 prescriptions, n (%) | 3,644 (44.7%) | 9,473 (18.7%) | <.001* | 4,410 (61.7%) | 7,983 (34.9%) | <.001* | 18,245 (30.3%) | 15,471 (14.2%) | <.001* | 50,973 (30.9%) | 9,856 (18.2%) | <.001* |
| **Combination therapy with ≥2 distinct AD treatments, n (%)** | **4,393 (53.8%)** | **12,468 (24.6%)** | **<.001*** | **4,533 (63.5%)** | **6,735 (29.5%)** | **<.001*** | **23,717 (39.4%)** | **16,324 (15.0%)** | **<.001*** | **64,723 (39.2%)** | **10,644 (19.6%)** | **<.001*** |
| **Topical treatments, n (%)** | **7,743 (94.9%)** | **46,450 (91.8%)** | **<.001*** | **5,621 (78.7%)** | **16,168 (70.8%)** | **<.001*** | **53,759 (89.2%)** | **95,805 (87.7%)** | **<.001*** | **143,734 (87.1%)** | **45,552 (84.0%)** | **<.001*** |
| Topical antihistamines | 0 (0.0%) | 25 (0.0%) | - | 0 (0.0%) | 5 (0.0%) | - | 2 (0.0%) | 11 (0.0%) | 0.128 | 22 (0.0%) | 14 (0.0%) | 0.049* |
| Any topical corticosteroids (TCS) | 7,666 (93.9%) | 45,216 (89.4%) | <.001* | 5,572 (78.0%) | 15,892 (69.5%) | <.001* | 53,648 (89.0%) | 94,969 (87.0%) | <.001* | 143,208 (86.8%) | 44,898 (82.8%) | <.001* |
| TCS low potency | 3,685 (45.2%) | 16,454 (32.5%) | <.001* | 2,339 (32.7%) | 5,574 (24.4%) | <.001* | 26,287 (43.6%) | 36,928 (33.8%) | <.001* | 68,128 (41.3%) | 15,054 (27.8%) | <.001* |
| TCS medium potency | 6,347 (77.8%) | 32,888 (65.0%) | <.001* | 4,706 (65.9%) | 12,358 (54.1%) | <.001* | 40,945 (67.9%) | 68,759 (63.0%) | <.001* | 106,486 (64.5%) | 33,292 (61.4%) | <.001* |
| TCS high potency | 2,813 (34.5%) | 13,726 (27.1%) | <.001* | 1,050 (14.7%) | 3,297 (14.4%) | 0.573 | 6,503 (10.8%) | 13,035 (11.9%) | <.001* | 20,718 (12.6%) | 8,941 (16.5%) | <.001* |
| Topical calcineurin inhibitors (TCI) | 963 (11.8%) | 6,880 (13.6%) | <.001* | 502 (7.0%) | 1,842 (8.1%) | 0.004* | 1,693 (2.8%) | 5,116 (4.7%) | <.001* | 4,840 (2.9%) | 3,741 (6.9%) | <.001* |
| **Systemic antihistamines^1^** | **5,933 (72.7%)** | **11,262 (22.3%)** | **<.001*** | **6,123 (85.7%)** | **7,023 (30.7%)** | **<.001*** | **42,416 (70.4%)** | **21,488 (19.7%)** | **<.001*** | **116,039 (70.3%)** | **12,044 (22.2%)** | **<.001*** |
| ≥ 1 sedating antihistamine | 3,466 (42.5%) | 9,078 (17.9%) | <.001* | 2,226 (31.2%) | 4,364 (19.1%) | <.001* | 16,564 (27.5%) | 14,916 (13.7%) | <.001* | 56,063 (34.0%) | 8,736 (16.1%) | <.001* |
| **Systemic corticosteroids (SC)^2^** | **2,200 (27.0%)** | **12,923 (25.5%)** | **0.006*** | **3,386 (47.4%)** | **9,702 (42.5%)** | **<.001*** | **12,630 (21.0%)** | **22,105 (20.2%)** | **<.001*** | **36,662 (22.2%)** | **14,896 (27.5%)** | **<.001*** |
| **Any systemic immunosuppressants (IMM)** | **51 (0.6%)** | **238 (0.5%)** | **0.064** | **15 (0.2%)** | **60 (0.3%)** | **0.437** | **96 (0.2%)** | **167 (0.2%)** | **0.751** | **314 (0.2%)** | **165 (0.3%)** | **<.001*** |
| Azathioprine | 7 (0.1%) | 36 (0.1%) | 0.650 | 3 (0.0%) | 11 (0.0%) | 0.834 | 14 (0.0%) | 22 (0.0%) | 0.677 | 30 (0.0%) | 24 (0.0%) | <.001* |
| Cyclosporine A | 8 (0.1%) | 68 (0.1%) | 0.397 | 6 (0.1%) | 27 (0.1%) | 0.447 | 17 (0.0%) | 35 (0.0%) | 0.665 | 54 (0.0%) | 37 (0.1%) | <.001* |
| Interferon gamma | 0 (0.0%) | 0 (0.0%) | 1.000 | 0 (0.0%) | 0 (0.0%) | 1.000 | 0 (0.0%) | 1 (0.0%) | - | 0 (0.0%) | 0 (0.0%) | 1.000 |
| Methotrexate | 28 (0.3%) | 123 (0.2%) | 0.098 | 7 (0.1%) | 25 (0.1%) | 0.796 | 45 (0.1%) | 91 (0.1%) | 0.546 | 181 (0.1%) | 100 (0.2%) | <.001* |
| Mycophenolate mofetil | 15 (0.2%) | 57 (0.1%) | 0.088 | 4 (0.1%) | 10 (0.0%) | 0.676 | 33 (0.1%) | 35 (0.0%) | 0.026* | 88 (0.1%) | 34 (0.1%) | 0.420 |
| **Intravenous immunoglobulin (IVIG)** | **110 (1.3%)** | **578 (1.1%)** | **0.109** | **76 (1.1%)** | **275 (1.2%)** | **0.338** | **705 (1.2%)** | **1,231 (1.1%)** | **0.432** | **1,448 (0.9%)** | **700 (1.3%)** | **<.001*** |
| **Montelukast sodium^3^** | **1,418 (17.4%)** | **6,956 (13.7%)** | **<.001*** | **3,028 (42.4%)** | **8,021 (35.1%)** | **<.001*** | **8,214 (13.6%)** | **15,542 (14.2%)** | **<.001*** | **25,059 (15.2%)** | **9,291 (17.1%)** | **<.001*** |
| **Phototherapy, n (%)** | **118 (1.4%)** | **245 (0.5%)** | **<.001*** | **39 (0.5%)** | **41 (0.2%)** | **<.001*** | **70 (0.1%)** | **103 (0.1%)** | **0.178** | **189 (0.1%)** | **133 (0.2%)** | **<.001*** |

**Notes:**

AD: Atopic dermatitis; SD: Standard deviation

1. The proportion of Medicaid and Commercial patients without comorbid asthma or allergies was 69.2% and 78.0% for dermatology, 17.4% and 29.1% for A/I, 64.8% and 73.8% for pediatrics, and 60.4% and 68.6% for other provider types.

2. The proportion of Medicaid and Commercial patients without comorbid asthma or allergies was 56.5% and 71.1% for dermatology, 7.9% and 17.3% for A/I, 49.3% and 63.2% for pediatrics, and 47.4% and 59.0% for other provider types.

3. The proportion of Medicaid and Commercial patients without comorbid asthma or allergies was 48.9% and 61.0% for dermatology, 7.5% and 15.8% for A/I, 42.9% and 56.9% for pediatrics, and 35.5% and 46.4% for other provider types.

*P-value<0.05. P-values were calculated using Chi-square tests for categorical variables, and Wilcoxon Mann-Whitney test for continuous variables.
